# Brassinosteroids: Biosynthesis, Signaling, and Hormonal Crosstalk as Related to Fruit Yield and Quality

**DOI:** 10.3390/plants14121865

**Published:** 2025-06-18

**Authors:** Divya Aryal, Fernando Alferez

**Affiliations:** Horticultural Sciences Department, Southwest Florida Research and Education Center, University of Florida/IFAS, Immokalee, FL 34142, USA; divyaaryal@ufl.edu

**Keywords:** crop production management, fruit maturation and quality, hormones, plant growth regulators

## Abstract

Brassinosteroids (BRs) are plant growth regulators (PGRs) with pleiotropic effects on plant growth and development. They play a role in seed germination, vegetative and reproductive growth, photosynthetic efficiency, vascular differentiation, fruit yield, quality, and resilience to biotic and abiotic stresses. They engage in crosstalk with other hormones like auxin, gibberellins, ethylene and abscisic acid, influencing all plant growth and development aspects. Studies on the effect of BRs on the reproductive growth of fruit crops are accumulating, given the potential of this PGR as a management tool in agriculture. This review explores the multifaceted roles of BRs in fruit crop maturation. From their biosynthesis and signal transduction pathways to their influence on fruit production, development, and maturation, we focus on the effect of this plant hormone on different aspects of fruit yield and quality, including fruit set and firmness, sugar accumulation, and fruit development. We address BRs’ interaction with different hormones at molecular and physiological levels in regulating these processes in climacteric and non-climacteric fruits. We also identify areas where knowledge is still lacking regarding hormonal crosstalk involving BRs in the regulation of developmental processes governing fruit quality and yield so knowledge generated can inform management decisions in fruit crop production.

## 1. Introduction

Plant hormones are important chemical messengers that can regulate different developmental processes and responses to stresses at low concentrations, producing cascade effects [1,2,3]. The role of hormones like auxin, gibberellins, cytokinin, ethylene, and abscisic acid and their interplay in regulating fruit maturation has been extensively studied in different fruit crops [4,5,6], while the role of brassinosteroids (BRs) has been relatively less explored. BRs are a relatively new member inducted into the plant hormone network. In 1970, Mitchell et al. reported the discovery of a new family of plant hormones, brassins, isolated from the pollen of rapeseed (*Brassica napus*). They identified brassins as a hormone because “they are specific translocatable organic compounds isolated from a plant and have induced measurable growth control when applied in minute amounts to another plant” [7]. Later, in 1979, single-crystal X-ray analysis identified an active ingredient in brassins as a steroidal lactone brassinolide (BL) [1]. BL is a steroid hormone structurally similar to animal steroid hormones like androgens, estrogens, and corticosteroids [1]. Later, in 1982, another steroid hormone, castasterone, was isolated from the insect galls of chestnut, *Castanea crenata* [8]. Since the first steroid hormone in plants was first extracted from brassica, the hormonal group was named brassinosteroid (BR). A study by [9] in *Arabidopsis thaliana* helped establish BRs as significant internal regulators in plant growth and development processes. To date, more than 70 BRs have been identified from different plant species [10]. However, only three, viz., Brassinolide, 24-Epibrassinolide, and 28-Homobrassinolide, have been found to be the most biologically active forms and are used in agriculture [11,12,13]. BRs are found in most plant tissues and organs, including pollen, seeds, fruits, shoot apex, young internodes, and roots [14]. BRs are involved in cell expansion, stem elongation, seed germination, vascular differentiation, stress toleration, and reproductive development, among other processes [12,15,16].

While BRs have been extensively studied in plants like *Arabidopsis thaliana*, their effects and applications in fruit crops remain relatively unexplored, and findings have not been critically reviewed, to the best of our knowledge, in fruit crops and woody plants. Research addressing their role in regulating fruit growth and development, interaction with other plant hormones, and their combined effects on fruit crop physiology and development remain underexplored. This paper aims to review current knowledge on BRs’ effects on fruit crop growth and development to identify knowledge gaps that may exist. Understanding the potential of BRs to interact with other hormones for practical application will allow for increased productivity and improved quality in agricultural management.

## 2. Chemical Structure of Brassinosteroids

**Figure 1:**
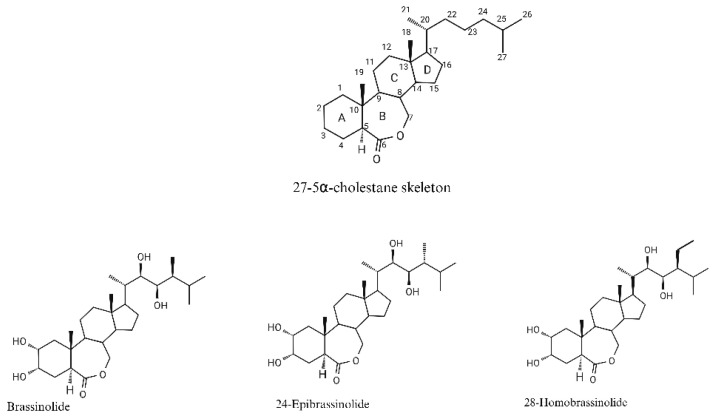
Chemical structure of common C_27_-5α-cholestane skeleton and some of the biologically active brassinosteroids.

BRs are polyhydroxylated plant steroid hormones having a common C_27_-5α-cholestane skeleton, C_28_-BRs: 5α-ergostane, and C_29_-BRs: 5α-stigmastane [17]. The variation among them depends on the type and position of the functionalities in the A/B rings and the side chain [8]. BRs can be classified as C_27_, C_28_, or C_29_ compounds based on the alkyl substitutions in the side chain [10,18]. For the proper functioning of the BRs, oxygen at the C_6_ position and hydroxyl group on the side chain at the C_22_ and C_23_ positions are essential. Some low-abundance BRs bear oxygen at C_3_ and additional ones at one or more of the C_2_, C_6_, C_22_, and C_23_ carbon atoms [19]. The chemical structures of BRs that are biologically active and used in horticulture are shown in Figure 1.

## 3. Biosynthesis, Transport, Signaling, and Homeostasis of Brassinosteroids

### 3.1. Biosynthesis

Endogenous BRs are assumed to be synthesized locally in ER membranes, as the enzymes involved in BR synthesis are localized in the ER membranes [14,20]. The precursor for their biosynthetic pathway is campesterol (CR), which is first converted into campestenol (CN). The biosynthesis is generally divided into CN-dependent and CN-independent pathways. CN-dependent pathways include early and late C-6 oxidation pathways [15], while CN-independent pathways include C-22 and C-23 hydroxylation pathways [21]. In the early C-6 pathway, CN is oxidized to form 6-oxocampestanol (6-oxoCN), and then, through a series of catalytic reactions, castasterone (CS) is formed. In the late C-6 oxidation pathway, CN is first hydroxylated to form 6-deoxocathasterone (6-deoxoCT), the non-oxidized form of CT, then undergoes catalytic reaction, same as the early C6 oxidation pathway, and forms CS in the final step, which is finally converted into Brassinolide (BL). The enzymes involved in CR catalysis to form BL are DWF4 (Dwarf 4), CPD (Constitutive photomorphogenesis and dwarfism), DET2 (De-etiolated 2), ROT3/CYP90D1 (Rotundifolia 3/Cytochrome P450 90D1), and CYP85A1/2 (Cytochrome P450 85A1/Cytochrome P450 85A2 [15,21,22].

### 3.2. Transport

Even though BRs are synthesized locally in ER membranes, receptors perceive BRs at the cell surface [14]. Thus, the endogenous BR transport is predicted to have short-distance movement within or between similar neighboring cells in each tissue [14,20]. Now, how the short-distance transportation occurs between the cells is a key question to be answered. Recently, a model for the plasmodesmata-mediated transportation of the BRs has been proposed [20].

### 3.3. Signal Transduction Pathways and BR Homeostasis

**Figure 2:**
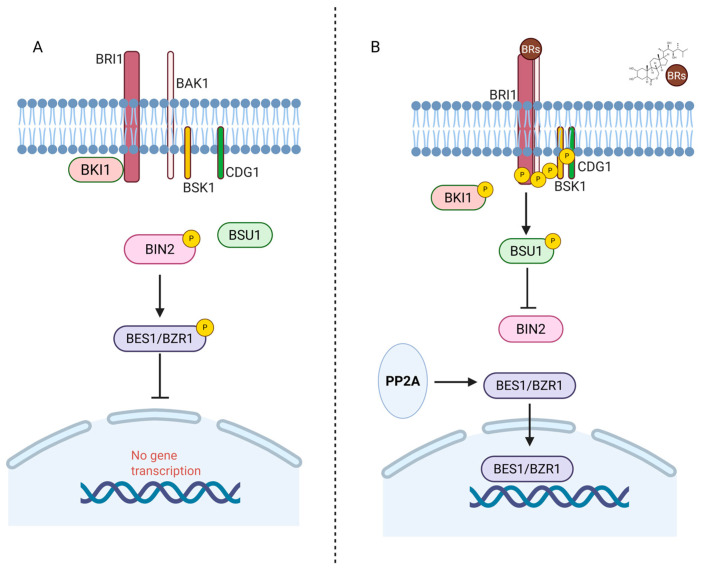
Brassinosteroid signaling pathway. (**A**) In the absence of BRs, the receptor complex remains inactive, and the kinase BIN2 phosphorylates the transcription factors BES1/BZR1, preventing their nuclear translocation and repressing BR-responsive gene expression. (**B**) In the presence of BRs, BRI1 forms an active receptor complex with co-receptors and initiates a phosphorylation cascade involving BSK1 and CDG1. This activates the phosphatase BSU1, which inactivates BIN2. Consequently, BES1/BZR1 are dephosphorylated by PP2A, accumulate in the nucleus, and activate BR-responsive gene expression. Image prepared.

BRASSINOSTEROID INSENSITIVE1 (BRI1), a leucine-rich repeat receptor kinase, plays a key role in BR signal transduction. In the absence of BRs, BRI1 is inactive due to its interaction with inhibitory proteins like BRI1 KINASE INHIBITOR1 (BKI1) or BOTRYTIS-INDUCED KINASE1 (BIK1). BRASSINOSTEROID INSENSITIVE2 (BIN2) is a negative regulator of the BRs signaling pathway. BIN2 phosphorylates two critical transcription factors, BRASSINAZOLE-RESISTANT1 (BZR1) and BRI1-EMS SUPPRESSOR1 (BES1), which are essential for the regulation of BR-responsive gene expression [23]. This phosphorylation inhibits the BR signaling cascade. However, in the presence of BRs, BRI1 phosphorylates BKI1 and disassociates from BKI1. BRI1 then interacts with its co-receptor, BRI1-ASSOCIATED RECEPTOR KINASE1 (BAK1, also known as SOMATIC EMBRYOGENESIS RECEPTOR-LIKE KINASE 3 or SERK3), resulting in trans-phosphorylation between BRI1 and the other SERK family members (SERK1, SERK2, and BKK1), activating the receptor complex [24]. Plasma membrane-associated copine proteins, specifically BONZAI (BON), ensure efficient BRI1-SERK complex formation and transphosphorylation [25]. Now, the activated BRI1-SERK receptor complex directly phosphorylates BR SIGNALING KINASES (BSKs) and CONSTITUTIVE DIFFERENTIAL GROWTH1 (CDG1), which, in turn, activates BRI1 SUPPRESSOR1 (BSU1) phosphatase. BSU1 dephosphorylates and inactivates BIN2. Thus, in the presence of BRs, BIN2 is deactivated, and BZR1 and BES1 are dephosphorylated. Then, BZR1 and BES1 are released, translocated into the nucleus, and allow the expression of BR-target genes essential for growth and development [13,23]. Hormone homeostasis is critical for normal plant growth and development [20,26,27]. BZR1 and BES1 regulate multiple BR-biosynthesis genes through a negative feedback loop that adjusts BR synthesis based on the levels of active BRs [26]. Thus, BZR1 has dual biological functions. When the BRs signal is activated, the BZR1 is unphosphorylated and accumulates in the nucleus, activating the gene expression for growth while inhibiting the expression of biosynthesis genes [28] (Figure 2).

## 4. Effects of Brassinosteroids on Fruit Crop Development

Fruit development, ripening, and maturation are very highly coordinated processes modulated by internal factors such as hormones and external factors such as environment. The interplay of hormones such as abscisic acid, gibberellins, and ethylene in regulating these processes has been studied for years [5]. However, how BRs may interact with these and other PGRs has been much less studied in fruit crops. BRs influence fruit development, from flower initiation to fruit maturation and ripening, by regulating cell division, expansion, elongation, and interaction with the other hormones [29,30,31].

### 4.1. Flowering, Fruit Set, and Maturation

In *Arabidopsis thaliana*, the FRIGIDA (FRI) protein regulates floral transition by activating the Flowering Locus C (FLC), a negative regulator of flowering, thereby delaying flowering [32]. Conversely, BRs suppress flowering inhibitor Flowering Locus C (FLC) and help plants transition from vegetative to reproductive growth [33]. Like in Arabidopsis, FRIGIDA-LIKE (SlFRLs) protein has been discovered in tomatoes, where SlFRLs interact with the BRs suppressor SlBIN2 and regulate early flowering [34]. However, the presence of BRs might reduce the SIBIN2 suppressive effect on SIFRLs and potentially delay flowering. This indicates that BRs have a dual effect on flowering and that this is a complex phenomenon. Moreover, the direct effect of the exogenous application of BRs on the flowering of fruit crops remains poorly studied. Further investigation needs to be performed to understand BRs potential to regulate flowering in different fruit crops, so it can become a profitable agricultural management practice.

More knowledge is available on fruit set, development, and maturation. In this regard, BR application during flowering has been reported to increase fruit set and crop yield in different fruit species, both climacteric and non-climacteric. It is well established that BRs improve yield in other crops. In Brazil, Br applications resulted in yield increases of 18, 22, and 83% in wheat, soybean, and common beans, respectively [35]. BRs also increased yield in wheat, rice, peanut, mustard, potato, and cotton [36]. When it comes to fruit crops, there are also accumulating examples of the effect of BRs inducing changes in fruit maturation and yield, regardless the fruit is classified as climacteric or non-climacteric: BRs treatment on litchi leaves before blossom increased fruit quality and commercial value by modifying peel quality characteristics of the fruit [37]. In passion fruit plants, the increase in the number of fruits after BRs treatment performed at flower anthesis was considerably greater than in the non-treated controls; in this study, BRs application led to an increase of 1 °Brix in the soluble solids content of the fruit juice, which is considered significant, because passion fruit pulp is very acid and any increase in the °Brix value index can contribute to juice quality [38]. In Navel oranges, foliar application of BRs at the flowering stage and 25 days afterward led to a notable increase in fruit set, but there was no modification in the internal fruit quality, probably because it was an early application, well before the fruit entered the maturation program. However, interestingly, there was a delay in leaf and fruit abscission [39] that resulted in less fruit drop, which may explain the greater yield. Furthermore, [40] observed a delay in the abscission of orange fruit when BRs were applied to the tree. A recent study has shown that even during postharvest, BR treatment may induce more anthocyanin accumulation in blood oranges while maintaining sugar levels that otherwise would decline during storage [41]. Further evidence of BRs’ impact on fruit set is seen in mango trees, where the application of BRs increased fruit set percentage, ultimately increasing fruit yield (kg/tree) [42]. The foliar application of BRs in apricots has also been shown to increase fruit set, yield, and physical and chemical characteristics of the fruit [43].

In the non-climacteric strawberry, internal BR content increases by the end of the fruit development, contributing to fruit maturation and quality [44]. Furthermore, when a low concentration of BRs was applied, the maturation process was accelerated [45]. Like in strawberries, endogenous BR levels increase during berry development at the onset of maturation in grapes. Applying BRs to grape berries at this stage has helped to promote maturation, while brassinazole application, an inhibitor of BR biosynthesis, delayed the process, proving the involvement of endogenous BRs [46,47]. In banana, the transcription factor MaBZR1/2 interacts with MaMPK14 to enhance the transcriptional repression of cell wall modification genes (MaEXP2, MaPL2, and MaXET5), slowing down softening, which is overcome at a later stage of development [48].

### 4.2. Fruit Size

Fruit size is an important attribute of fruit crops, influencing fruit yield [49] and, ultimately, value. Cell division and expansion drive fruit size increment over the developmental period [50,51,52]. Meanwhile, the application of BRs may boost the size and quantity of fruits [15]. BRs increase cluster and berry weights in grapes and size in different climacteric and non-climacteric fruits, including strawberries, mangoes, persimmons, apples, and cherries [42,44,53,54,55,56]. The direct involvement of this hormone on fruit size has been further shown recently in tomato at the molecular level, as BR-insensitive tomatoes show a reduction in fruit size and weight due to decreased cell size and number of cellular layers [57]. In the same study, fruit-specific genes like tomato sucrose transporter (SlSUT), tomato protein kinase (SlWEE1), tomato auxin transcription factor (SlIAA9), tomato cyclin-dependent kinases (SlCDKA1 and SlCDKB2), brassinolide responsive gene (GAox20), and tomato fruit weight gene (SlFW2.2) were deficient in the BR-insensitive tomatoes, demonstrating the importance of BR-signaling in fruit size [57]. However, how BR signaling affects these genes is still not well understood, and how BRs affect the fruit size in other fruits needs to be explored.

### 4.3. Fruit Firmness

The effect of BRs on softening seems to be disparate depending on the fruit and developmental stage. In climacteric fruits like mango, persimmon, and apple, exogenous BRs application at ripening advances the onset of ethylene biosynthesis and increases cell wall degradation enzyme activity, resulting in fruit softening [55,56,58]. In contrast, post-harvest treatment of BRs in the climacteric fruit peach has been shown to inhibit pectin degradation enzyme polygalacturonase (PG), pectin lyase1 (PL), and pectin methylesterase (PME), resulting in a delay of fruit softening, the release of ethylene, water-soluble pectins, and ionic soluble pectins [59]. BRs also inhibited the expression of pectin degrading genes (PME1/3, PpPG, PpARF2, and PpGAL2,/16)and reduced expression of PpBES1-5/6, transcription factors involved in BRs signaling; this resulted in fruit softening delay and increase in firmness in peach [59]. A similar effect has been observed in non-climacteric fruit like sweet cherries, where BR application at post-harvest reduced chilling injury, preserved structural integrity, and maintained fruit firmness. This, in turn, enhanced fruit quality and post-harvest longevity [60].

### 4.4. Sugar Accumulation

In climacteric fruits like mango, persimmon, and apple, pre-harvest exogenous BRs application increases the total soluble solids [55,56,58]. Like in climacteric fruits, in non-climacteric fruits such as strawberries, grapes, and sweet cherries, BR application increased total soluble solids and starch content [45,46,54]. However, in fruits like strawberries and sweet cherries, the sugar accumulation is higher in the earlier stage (white stage, strawberry; swollen stage, sweet cherry) and gradually decreases thereafter [54,61]. These observations indicate that application time for BR treatments is critical to achieving significant results for growers. Interestingly, increased sugar accumulation may be due to increased photosynthesis activity. In this sense, BRs elevate PSII efficiency, stomatal conductance, and carbon fixation by promoting the activity of RuBP (ribulose 1,5-bisphosphate) carboxylase, thus improving photosynthetic efficiency [62].

### 4.5. Color Development

In climacteric fruits like mango, persimmon, and apple, pre-harvest exogenous BR application increases anthocyanin production [55,56,58]. Similarly, BRs enhanced fruit color development and anthocyanin accumulation during maturation in non-climacteric fruit like citrus, strawberry, and grapes [41,45,53,63]. In strawberries, mRNA expression levels of FaBRI1 increased rapidly during the transition from white to initial red stages, and downregulation of FaBRI1 expression markedly retarded strawberry fruit red coloring, suggesting the BR’s involvement in fruit color development [44]. However, how color development is influenced by the downregulation of FaBRI1 must be explored in relation to how BRs interact with other plant hormones in regulating this process.

Together, these findings indicate that BR effects may depend not only on the fruit but also on the developmental and maturation stages. All in all, it seems clear that BRs as an exogenous treatment can be an agricultural management tool to increase fruit yield and quality attributes, but data indicates that the timing of application plays an important role in order to achieve maximum results. This needs to be determined for each crop. Table 1 summarizes the effects of BR application on different aspects of fruit maturation and quality.

## 5. BRs Interaction with Other Plant Hormones During Fruit Development, Ripening, and Maturation

**Figure 3:**
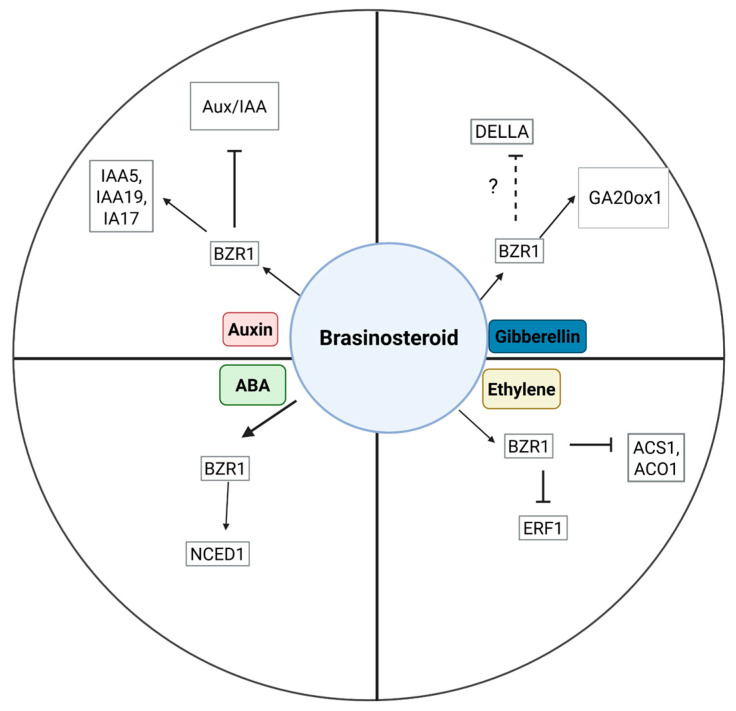
Diagram depicting BR’s interactions with other plant growth regulators at the molecular level of biosynthesis and signaling, including ABA, ethylene, auxins, and gibberellins during biosynthesis and signaling. Discontinuous line indicates a proposed effect but not yet fully demonstrated.

BRs are not transported over long distances within the plant [64]. However, they may impact the transport and synthesis of other hormones, like auxin, gibberellins, cytokinin, and ethylene, contributing to coordinated growth responses in different plant tissues [14,15,30,65]. Fruit development is regulated by a complex network of phytohormonal interactions, with auxin, gibberellins (GAs), abscisic acid (ABA), and ethylene playing major roles [66]. BRs interact with these hormones at various stages of fruit development, influencing fruit set, size, quality, maturation, and ripening by modulating hormone signaling [67,68] (Figure 3).

For instance, in apples, exogenous BR application increased growth by upregulating auxin and gibberellin biosynthesis genes and downregulating genes encoding negative regulators of auxin and gibberellin signal transduction, reinforcing the relationship between BRs, auxins, and gibberellins pathways [30]. Understanding the crosstalk between BRs and other plant hormones is crucial, as their effects vary among species and will dictate agricultural management. It is known that BR signaling components primarily interact with the signaling elements of other hormones at the transcriptional level [65,69]. However, a key challenge is determining how BR signaling regulates different BR-controlled processes, such as fruit development, in coordination with other hormones.

### 5.1. Auxin

Auxin controls many aspects of fruit development, primarily involving cell division and enlargement [70,71]. Auxin Responsive Factors (ARFs) are mostly involved in the maturation of reproductive organs in papaya, tomato, litchi, fig [72,73,74], and anthocyanin biosynthesis in apples [75]. Various studies have provided evidence of BRs and auxin signaling interdependency in cell expansion, hypocotyl elongation, and vascular bundle development [15,76,77]. Auxin interaction with other hormones mainly involves Aux/IAA and Auxin Responsive Factor (ARF) proteins [71]. In this sense, BRs have been shown to induce the expression of auxin-responsive genes IAA5, IAA19, and IAA17 during root development. These genes were downregulated in the BRs biosynthetic mutant de-etiolated2 (det2), indicating that BRs are required for auxin-dependent gene expression [78]. Moreover, the BIN2 kinase involved in BRs signaling phosphorylates and inactivates the ARF repressor in photomorphogenesis processes [79]. The possibility of a similar interplay between auxin and BRs during fruit development deserves to be explored.

### 5.2. Gibberellin

Physiological and molecular studies have shown that BRs and gibberellic acid (GA) signaling pathways interact to regulate cell elongation and growth [80]. In *Arabidopsis thaliana*, the BRs regulation of GA biosynthesis occurs through the BRs signal-responsive transcription factors BES1 and BZR1, which bind promoters of GA biosynthetic genes [80]. In some non-climacteric fruits, such as grapes and sweet cherries, the exogenous application of BRs and GA combination improves fruit quality attributes like soluble solids, titratable acidity, and anthocyanin content more effectively than their individual application [81,82]. Other fruits like pears also increase sugar content when BRs are applied together with GA [83]. Interestingly, GA application delays fruit ripening and maturation in other fruits, both climacteric and non-climacteric [5,84,85]. Together, all these observations show that interaction between both PGRs is complex and species-dependent. A possible mode of action for BRs in the interaction with GA promoting maturation could be via decreasing DELLA protein levels, a negative regulator of the gibberellin pathway, as observed during ovule formation in tomatoes [86].

### 5.3. Cytokinin

The cytokinin (CK), namely N 6-(Δ2-isopentenyl)-adenine (iP), concentration increases during ripening in grapes, strawberries, and tomatoes [87]. Moreover, the change in the iP content correlates with an increase in the expression of Isopentenyl transferases (IPTs) in the fruit, suggesting a role of IPT in cytokinin synthesis and, thereby, in ripening [87]. BR and cytokinin interplay has been documented for plant growth and CK-induced anthocyanin production [69]. Interestingly, it has been pointed out that BR–CK cross-talk may contribute to the modification of source/sink relations, improving crop yield and stress responses. However, studies on CK-BR interaction have been mostly made in model plants and cereals, and studies in fruit development are needed.

### 5.4. Ethylene

Ethylene biosynthesis is tightly regulated and promotes ripening in climacteric fruits [88]. BRs, however, possibly play a key role in modulating the process. Ethylene biosynthesis is primarily regulated by two enzymes: 1-aminocyclo propane-1-carboxylic (ACC) synthase (ACS) and ACC oxidase (ACO) [88]. In pears and apples, the BR responsive transcription factor BRASSINAZOLE-RESISTANT 1(PuBZR1) binds to the promoter of PuACO1 and PuACS1 and directly suppresses its transcription, thus preventing ethylene biosynthesis. Alternatively, PuBZR1 is bound to the Ethylene Response Factors (PuERF) promoter, a transcription factor that activates ACO1 and ACS and prevents ethylene biosynthesis [89]. A similar mechanism has been observed in bananas, where BZR binds to BRRE motifs MaACS1 and MaACO13/14 and suppresses ripening [90]. Therefore, when endogenous BR levels decline as ripening proceeds in both banana and pears, BZR1 expression declines, which weakens the suppression of BZR1 on the transcription of ACO1 and ACS1, leading to a burst of ethylene production and fruit ripening [89,90].

Interestingly, exogenous application of BRs promoted ripening, possibly due to the activation of MaACS1 and MaACO13/14 in bananas [90], whereas exogenous BRs application during storage inhibited apple and pear ripening, thus maintaining firmness [89]. Similarly, BR-treated jujube (*Zizyphus jujube*) fruit showed a reduction in ethylene production during storage, thus increasing shelf-life [91]. In contrast, BR treatment at storage in persimmon promoted ripening ethylene biosynthesis and cell wall degrading enzymes [56]. In tomatoes, treatment with BRs promoted the expression of SlACS and SlACO genes involved in ethylene biosynthesis [31].

In non-climacteric fruit, the mechanisms of BR–ethylene interaction are less studied. In non-climacteric fruit, ethylene receptor expression seems to increase during maturation, indicating a role for ethylene in maturation [92,93,94,95], and interaction with other hormones such as ABA is well established [4,5]. In strawberries, endogenous BR levels increase during the late developmental stages, and the mRNA expression levels of FaBRI1 increase rapidly during the transition from white to initial red stages, supporting the involvement of BRs in fruit maturation [44]. In the same study, when exogenous BRs and BRs inhibitors were applied, they promoted and inhibited maturation, respectively, which further supports this notion [44]. Similarly, in grapes, endogenous BRs increase at the onset of maturation, and exogenous BR application promotes maturation, while BR inhibitors inhibit the process [46]. In sweet orange, BR application promoted internal fruit maturation [96], but its interplay with ethylene has not been studied. All in all, how BRs interact with ethylene in non-climacteric fruit still needs to be explored at molecular and physiological levels.

### 5.5. Abscisic Acid

Abscisic acid (ABA) plays a regulatory role in fruit development and maturation in both climacteric and non-climacteric fruits [5,97,98]. This PGR controls the expression of genes involved in color and texture and interacts with ethylene, sugars, and other molecules [5,97]. The involvement of Brs in regulating ABA biosynthesis has been observed in tomatoes during chilling stress, where overexpression of the BR biosynthesis gene DWARF (DWF) and BR signaling gene BRASSINAZOLE-RESISTANT1 (BZR1) increased ABA levels by regulating the expression of the ABA biosynthesis gene 9-CIS-EPOXYCAROTENOID DIOXYGENASE1 (NCED1) [99]. In citrus, increased ABA levels during maturation modulated defense responses and interacted with other hormones like ethylene, influencing various biochemical and physiological events associated with fruit maturation [100]. However, the role of BRs in modulating these responses has not been explored yet. Understanding ABA and BR’s crosstalk and their combined practical application in fruit ripening is crucial and can potentially improve fruit quality. Future research should focus on untangling BR and ABA interactions.

## 6. Conclusions

The ability of BRs to regulate fruit growth and development makes this PGR an invaluable tool in horticulture, offering farmers a natural means to enhance fruit production and quality in various species. BRs have a positive or negative impact on the fruit development process depending on fruit type, stage of maturation, and time of application. Moreover, the interaction of BRs with other PGRs may improve fruit quality. Effectively determining these interactions will be of paramount importance for BR adoption in different agricultural commodities.

## Figures and Tables

**Table 1:** Effects of BRs application and timing on different fruit quality parameters in climacteric and non-climacteric fruit.

Crop	Effect of BR Application	Time of Application	Reference
Climacteric			
Passion fruit	Increase in number of fruits, increase in soluble solids content	At flower anthesis	[38]
Mango	Increase in fruit set and quality in drought stress conditions Fruit softening, increase in sugars	Flower bud induction and differentiation, full bloom, and beginning of fruit set Hard mature fruit stage	[42,58]
Apricot	Increase in fruit set, yield, total soluble sugars, and vitamin C	At swelling bud stage, at the balloon stage, just after fruit set, and one month before harvest	[43]
Persimmon	Fruit softening, increase in sugars	At preclimacteric stage	[56]
Galaxy apple	Increase in soluble solids, color, and antioxidant activities	Every 15–21 days, starting 40 days after full bloom	[55]
Peach	Delay of fruit softening	After harvest	[59]
Non-climacteric			
Litchi	Fruit cracking reduction	Before flower anthesis	[37]
Navel oranges	Increase in fruit set, delay in organ abscission	At flower anthesis and 25 days afterward	[39]
Blood oranges	Increase in anthocyanin content, delay in sugar decrease during storage	After harvest	[41]
Strawberry	Accelerate maturation Increase in sugars, growth acceleration	Big-green stage 15 days after anthesis (fast fruit growing stage) and at the end of maturation	[44,45]
Grape berries	Accelerate maturation. Sugar accumulation	Onset of color change	[46]
Sweet cherries	Reduction in chilling injury, fruit firmness is maintained Increase in fruit size and anthocyanin content	After harvest Swollen bud stage	[54,60]
